# Effect of different corticosteroid regimes for hospitalised patients with exacerbated COPD: pooled analysis of individual participant data from the REDUCE and CORTICO-COP trials

**DOI:** 10.1186/s12931-021-01745-5

**Published:** 2021-05-21

**Authors:** Pradeesh Sivapalan, Jonas Rutishauser, Charlotte Suppli Ulrik, Jörg D. Leuppi, Lars Pedersen, Beat Mueller, Josefin Eklöf, Tor Biering-Sørensen, Vibeke Gottlieb, Karin Armbruster, Julie Janner, Mia Moberg, Therese S. Lapperre, Thyge L. Nielsen, Andrea Browatzki, Alexander Mathioudakis, Jørgen Vestbo, Philipp Schüetz, Jens-Ulrik Jensen

**Affiliations:** 1 Section of Respiratory Medicine, Department of Internal Medicine, Herlev and Gentofte Hospital, University of Copenhagen, Hellerup, Denmark; 2 Department of Medicine, Clinical Trial Unit, Kantonsspital Baden, 4054 Baden, Switzerland; 3 Faculty of Medicine, University of Basel, 4001 Basel, Switzerland; 4 Department of Respiratory Medicine, Copenhagen University Hospital-Hvidovre, Hvidovre, Denmark; 5 University Clinic of Medicine, Kantonsspital Baselland, 4410 Liestal, Switzerland; 6 Department of Respiratory Medicine, Bispebjerg University Hospital, Copenhagen, Denmark; 7 Department of Cardiology, Herlev and Gentofte Hospital, University of Copenhagen, Hellerup, Denmark; 8 Department of Biomedical Sciences, Faculty of Health and Medical Sciences, University of Copenhagen, Copenhagen, Denmark; 9 Department of Respiratory Medicine, Antwerp University Hospital, and Laboratory of Experimental Medicine and Pediatrics, University of Antwerp, Antwerp, Belgium; 10 Department of Respiratory and Infectious Diseases, Frederiksund and Hillerød Hospital, University of Copenhagen, Copenhagen, Denmark; 11 The North West Lung Centre, Wythenshawe Hospital, Manchester University NHS Foundation Trust, Manchester, UK; 12 Division of Infection, Immunity and Respiratory Medicine, School of Biological Sciences, The University of Manchester, Manchester Academic Health Science Centre, Manchester, UK; 13 Medical University Department, Kantonsspital Aarau, 5001 Aarau, Switzerland; 14 Department of Internal Medicine, Zealand University Hospital, University of Copenhagen, 4000 Roskilde, Denmark

**Keywords:** COPD, Exacerbation, Corticosteroids, Mortality, Days alive and out of hospital, Intensive care unit

## Abstract

**Background:**

Systemic corticosteroid administration for severe acute exacerbations of COPD (AECOPD) reduces the duration of hospital stays. Corticosteroid-sparing regimens have showed non-inferiority to higher accumulated dose regimens regarding re-exacerbation risk in patients with AECOPD. However, it remains unclear whether 14-day or 2–5-day regimens would result in shorter admission durations and changes in mortality risk. We explored this by analysing the number of days alive and out of hospital based on two randomised controlled trials with different corticosteroid regimens.

**Methods:**

We pooled individual patient data from the two available multicentre randomised trials on corticosteroid-sparing regimens for AECOPD: the REDUCE (*n* = 314) and CORTICO-COP (*n* = 318) trials. In the 14-day regimen group, patients were older, fewer patients received pre-treatment with antibiotics and more patients received pre-treatment with systemic corticosteroids. Patients randomly allocated to the 14-day and 2–5-day regimens were compared, with adjustment for baseline differences.

**Results:**

The number of days alive and out of hospital within 14 days from recruitment was higher for the 2–5 day regimen group (mean 8.4 days; 95% confidence interval [CI] 8.0–8.8) than the 14-day regimen patient group (4.2 days; 95% CI3.4–4.9; *p* < 0.001). The 14-day AECOPD group had longer hospital stays (mean difference, 5.4 days [standard error ± 0.6]; *p* < 0.0001) and decreased likelihood of discharge within 30 days (hazard ratio [HR] 0.5; 95% CI 0.4–0.6; *p* < 0.0001). Comparing the 14-day regimen and the 2–5 day regimen group showed no differences in the composite endpoint ‘death or ICU admission’ (odds ratio [OR] 1.4; 95% CI 0.8–2.3; *p* = 0.15), new or aggravated hypertension (OR 1.5; 95% CI 0.9–2.7; *p* = 0.15), or mortality risk (HR 0.8; 95% CI 0.4–1.5; *p* = 0.45) during the 6-month follow-up period.

**Conclusion:**

14-day corticosteroid regimens were associated with longer hospital stays and fewer days alive and out of hospital within 14 days, with no apparent 6-month benefit regarding death or admission to ICU in COPD patients. Our results favour 2–5 day regimens for treating COPD exacerbations. However, prospective studies are needed to validate these findings.

**Supplementary Information:**

The online version contains supplementary material available at 10.1186/s12931-021-01745-5.

## Background and rationale

Current guidelines recommend that systemic corticosteroids should be used to treat severe acute exacerbations of chronic obstructive pulmonary disease (AECOPD) [1]. A meta-analysis of randomised controlled trials (*n* = 1700) assessed the effectiveness of systemic corticosteroids compared to placebo in treating COPD exacerbations. Most of the studies from the meta-analysis were based on hospitalised patients. The meta-analysis showed that corticosteroids shorten hospital stays by approximately 1 day and have a moderately positive effect on pulmonary function tests and 30-day re-exacerbation rate, but do not seem to have any influence on survival [2]. The effects of systemic corticosteroids in treating AECOPD are temporary, lasting only 3–5 days. The risk of admission to an intensive care unit (ICU) or re-exacerbations after the first month appears to be unaltered [2, 3]. Corticosteroids affect the expression of various genes, especially those regulating the innate and the adaptive immune system [4], leading to a variety of beneficial and harmful effects [5–8] such as increasing the risk of acute or chronic infections [9, 10], the onset or aggravation of diabetes mellitus [11, 12], and osteoporotic fractures[9]. Therefore, minimising the unnecessary exposure of patients with chronic obstructive pulmonary disease (COPD) exacerbations to systemic corticosteroids is crucial. To date, two RCTs have investigated how such unnecessary exposure can be minimised, without attenuating the documented beneficial effects [13, 14]. In the REDUCE trial, patients hospitalised for AECOPD were randomly assigned to either 14 days (standard) or 5 days (intervention) of treatment with 40 mg of daily prednisone. The CORTICO-COP trial randomly assigned patients to 5-day (standard) or eosinophil-guided (intervention) therapy, with the latter regimen resulting in a median of 2 days of treatment with prednisolone.

However, it remains unclear whether 14-day or 2–5-day regimens would result in shorter admission durations and changes in mortality risk. We explored this by analysing the number of days alive and out of hospital based on two trial populations with different dosing regimens.

## Material and methods

### Study participants

We studied individual patient data from two investigator-initiated non-inferiority trials of systemic corticosteroids for exacerbated COPD. The first study was the REDUCE trial, a multicentre randomised controlled non-inferiority study (*n* = 314) comparing a short-term (5 days) with a conventional (14 days) systemic corticosteroid treatment. From March 2006 through February 2011, consecutive patients with AECOPD were screened for eligibility at the emergency departments of five Swiss teaching hospitals. Inclusion criteria were exacerbation of COPD as defined by the presence of at least two of the following: change in baseline dyspnoea, cough, or sputum quantity or purulence, age older than 40 years, and a smoking history of 20 pack-years or more. Exclusion criteria were a history of asthma, ratio of forced expiratory volume in 1 s (FEV_1_) to forced vital capacity (FVC) greater than 0.70 as evaluated by bedside post-bronchodilator spirometry prior to randomisation, radiological diagnosis of pneumonia, estimated expected survival of less than 6 months due to severe comorbidity, pregnancy or lactation, prior inclusion in the trial, and inability to provide written informed consent. Written informed consent was obtained from all patients before randomisation. The follow-up period was 6 months [13].

The other study was the CORTICO-COP trial, a nationwide multicentre prospective trial (*n* = 318) investigating eosinophil-guided corticosteroid treatment for AECOPD [14]. All consecutive patients admitted to the wards of the participating sites were eligible if they were included within 24 h of admission, were aged at least 40 years old, had known airflow limitation (defined as post-bronchodilator FEV_1_/FVC ratio ≤ 0.70), and a specialist-verified diagnosis of COPD based on stable disease-state data. Exacerbations were defined according to the consensus definition described by the Global Initiative for Chronic Obstructive Lung Disease (GOLD) committee: acute worsening of respiratory symptoms that results in additional therapy. Exclusion criteria included any history of asthma (self-reported or physician-diagnosed), life expectancy of less than 30 days, severe COPD exacerbation requiring invasive ventilation or admission to an ICU, allergy to systemic corticosteroids, severe mental illness that could not be controlled by medication, people detained under the act on the use of coercion in psychiatry, severe language difficulties or the inability to provide written informed consent, pregnancy or lactation, systemic fungal infections, or patients receiving more than 10 mg of maintenance systemic corticosteroids daily. Written informed consent was obtained from patients before randomisation. Patients could only participate in the trial once.

### Interventions

In the REDUCE trial, patients were randomised to either: (a) 5 days of 40 mg daily systemic prednisone, followed by 9 days of placebo or (b) 14 days of 40 mg daily systemic prednisone.

In the CORTICO-COP trial, all patients received 80 mg of methylprednisolone on day 1, followed by either: (a) 37.5 mg of daily prednisolone for 4 days or (b) prednisolone only on the days when peripheral blood eosinophil count was ≥ 300 cell/microliter Most patients' first eosinophil count was recorded prior to their first dose of corticosteroids.

**Fig. 1:**
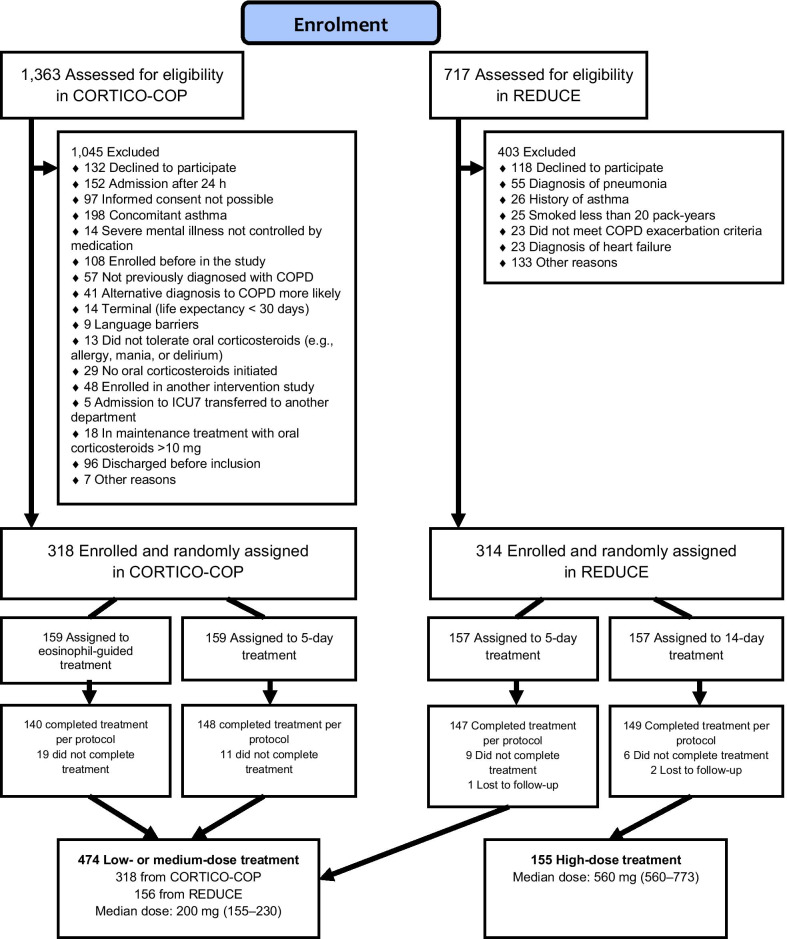
Flow chart showing the study cohort

Apart from prednisolone, all participants received additional nebulized short-acting beta-2 agonists and short-acting antimuscarinic agents as needed while hospitalised. Physiotherapy, supplemental oxygen, and ventilatory support were administered according to American Thoracic Society/European Respiratory Society guidelines. We compared the 14-day prednisone group with the 2–5 day prednisone group (Fig. 1). The 2–5 days prednisone treatment group was considered the reference group.

### Outcome measures

The primary outcome was days alive and out of hospital within 14 days after recruitment. We also analysed the following short- and long-term secondary outcomes: (i) length of hospital stay, (ii) rate of discharge within 30 days, (iii) all-cause mortality during a 6-month follow-up period, (iv) combined endpoint ‘ICU admission and all-cause mortality’ within 6 months, and (v) newly diagnosed or aggravation of pre-existing hypertension.

### Statistical methods

Continuous normally distributed variables are presented as means ± standard deviations (SD), non-normally distributed data are presented as medians with interquartile ranges. Categorical variables are presented as counts and proportions. Lengths of hospital stay are reported as means ± SD. Cox proportional hazards regression models were used to assess the association between corticosteroid dose and all-cause mortality during the 6-month follow-up period, as well as time to discharge within 30 days. The multivariable model was adjusted for possible confounding variables, including age, sex, smoking status, pre-treatment with antibiotics, and pre-treatment with corticosteroids. Logistic regression models were used to analyse the outcomes “death or admission to ICU during the 6-month follow-up period” and “new or aggravated hypertension” and adjusted for the same confounding variables. Statistical significance was defined as a *p*-value ≤ 0.05. Statistical analyses were performed using SAS version 9·4 (SAS Institute, Inc., Cary, NC, USA) and the statistical software R (version 3.6.3).

## Results

The mean age of the 629 patients included in this study was 72 ± 10 years, and 52.5% of them were male. The baseline demographic data for the two trials were not similar. In the CORTICO-COP trial, the mean age was higher, more patients were female, and pre-treatment with antibiotics was more common. In contrast, patients in the REDUCE trial were more likely to have had pre-treatment with systemic corticosteroids (Additional file 1: Table 1). In the 14-day regimen group, patients were older, fewer received pre-treatment with antibiotics and more patients received pre-treatment with systemic corticosteroids. In addition, there were slight differences in blood pressure between the groups at baseline (Table 1).

**Table 1:** Baseline characteristics of study participants from the primary trials

	14-day OCS regimen	2–5 days OCS regimen	*p* value
	*n* = 155	*n* = 474	
Age in years, mean (SD)	72.4 (10.4)	63.3 (10.2)	0.0004
Women, no. (%)	69 (46.3)	224 (47.9)	0.74
Index steroid dose in mg, median (IQR)	560 (560–560)	200 (155–230)	< 0.0001
Smokers, no. (%)			
Current	62 (40)	181 (38.2)	0.69
Past smoker	93 (60)	287 (60.5)	0.90
Never smoked	0 (0.0)	6 (1.3)	0.16
Medical Research Council dyspnoea scale, no. (%)			
1	4 (2.8)	12 (2.6)	0.90
2	14 (9.8)	37 (8.0)	0.51
3	15 (10.5)	116 (25.2)	0.0002
4	43 (30.1)	144 (31.3)	0.78
5	67 (46.9)	151 (32.8)	0.0023
Pre-treatment with antibiotics, no. (%)*	21 (13.5)	118 (24.9)	0.0031
Pre-treatment with systemic glucocorticoids, no. (%)**	24 (15.5)	34 (7.2)	0.0019
Pack years, median (IQR)	45 (30–60)	45 (30–58)	0.40
FEV_1_ baseline, median (IQR) % predicted	28.1 (21.0–40.8)	30.0 (22.2–40.0)	0.40
FEV_1_ day 30. median (IQR) % predicted	43.1 (32.2–60.0)	41.0 (28.5–55.0)	0.14
Clinical values, median (IQR)			
Blood pressure, mm Hg			
Systolic blood pressure	138 (124–158)	130 (118–145)	0.0019
Diastolic blood pressure	80 (70–87.5)	72 (65–84)	0.0004
Heart rate, beats per min	90 (79–105)	89 (80–101)	0.81
Saturation with supplemental oxygen %	95 (92–97)	95 (93–96)	0.94

*OCS* oral corticosteroids, *FEV*_*1*_ forced expiratory volume in 1 s, *SD* standard deviation, *IQR* interquartile range

*Data refer to treatment for the index acute COPD exacerbation

**Data refer to treatment prior to index acute COPD exacerbation, defined as daily therapy over 2 days or more directly before the day of inclusion

**Fig. 2:**
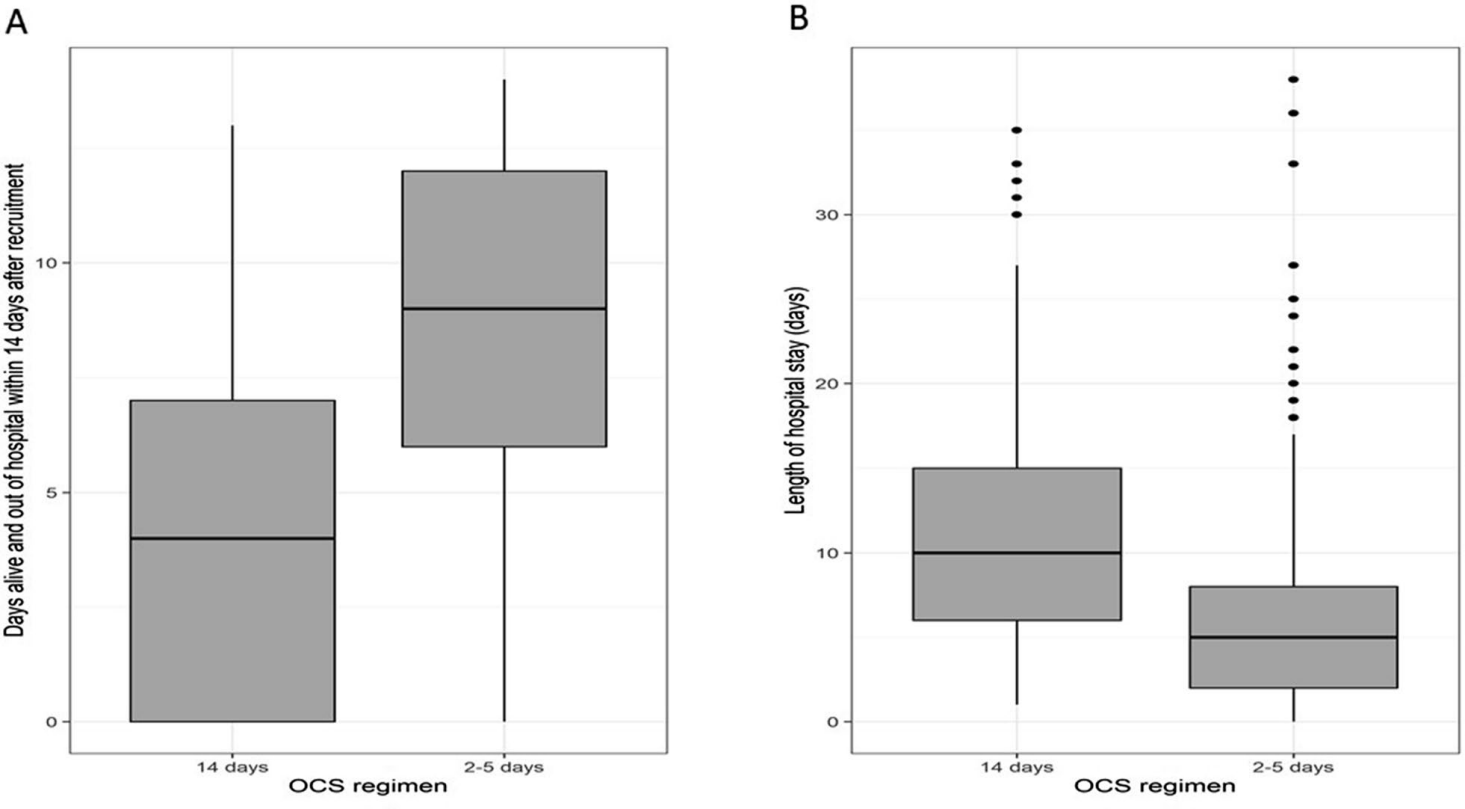
**A** Days alive and out of hospital within 14 days, **B** Length of hospital stay

**Fig. 3:**
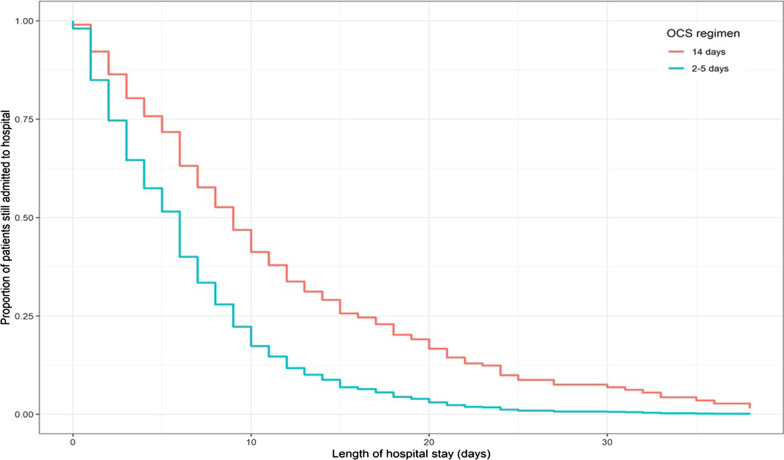
Time to discharge within 30 days

As shown in Table 2, the number of days alive and out of hospital within 14 days was greater for the low–medium (mean 8.4 days; 95% confidence interval [CI] 8.0–8.8) than the 14-day regimen group (mean 4.2 days; 95% CI 3.4–4.9; *p* < 0.001; Fig. 2A). The 14-day regimen group had longer hospital stays (mean difference 5.4 days [SE ± 0.6]; *p* < 0.0001; Fig. 2B), and lower likelihood of discharge within 30 days (hazard ratio [HR] 0.5; 95% CI 0.4–0.6; *p* < 0.0001; Fig. 3). An adjusted Cox regression analysis revealed no difference between the two groups in mortality risk during the 6-month follow-up period (HR 0.8; 95% CI 0.4–1.5; *p* = 0.45). Adjusted logistic models showed there were no differences between the two groups in risk of ‘death or admission to an ICU’ (odds ratio [OR] 1.4; 95% CI 0.8–2.3]; *p* = 0.15) or new/aggravated hypertension (OR 1.5; 95% CI 0.9–2.7; *p* = 0.15) during the 6-month follow-up period.

**Table 2:** Analysis of outcomes

	2–5 day regimen	14-day regimen	*p*-value
	*n* = 474	*n* = 155	
*Primary outcome measure*			
Days alive and out of hospital within 14 days, Mean (95% CI)	8.3 (7.9–8.7)	4.2 (3.6–4.9)	< 0.001
*Secondary outcome measures*			
Cumulative median dose corticosteroids (mg)			
over 6-month follow-up period (IQR)	322.5 (200–605)	560 (560–773)	
Death during 6-month follow-up period			
Adjusted HR (95% CI)^a^	Reference	0.8 (0.4–1.5)	0.45
Death or admission to ICU during 6-month follow-up period			
Adjusted OR (95% CI)^b^	Reference	1.4 (0.9–2.3)	0.25
New or aggravated hypertension^b^			
Adjusted OR (95% CI)	Reference	1.5 (0.9–2.7)	0.15
Length of hospital stay^c^			
Adjusted mean days (SE)	Reference	+ 5.4 (± 0.6)	< 0.0001
Probability of discharge within 30 days^a^			
Adjusted HR (95% CI)	Reference	0.6 (0.5–0.70)	< 0.0001

*CI* confidence interval, *HR* hazard ratio, *OR* odds ratio, *ICU* intensive care unit, *SE* standard error

^a^Based on Cox proportional hazards model adjusted for age, sex, smoking status, pre-treatment with antibiotics, pre-treatment with corticosteroids, and Medical Research Council dyspnoea scale

^b^Based on logistic model adjusted for age, sex, smoking status, pre-treatment with antibiotics, pre-treatment with corticosteroids, and Medical Research Council dyspnoea scale

^c^Based on means and standard error

As a sensitivity analysis, we looked at the length of hospital stay and mortality rates for the 5-day regimen compared to the 14-day regimen. The 5-day regimen resulted in a shorter length of stay (mean difference, 5.4 days [SE ± 0.6]; *p* < 0.0001). The mortality rates in both groups were similar during the 6-month follow-up period (HR 0.8 [0.4–1.7]; *p* = 0.58; Additional file 1: Table 2).

## Discussion

This two-study meta-analysis showed that patients randomly allocated to the 2–5-day corticosteroid dose group had more days alive and out of hospital within 14 days after recruitment than those randomly allocated to the 14-day dose group. In addition, data recorded for the lengths of hospital stay and time to discharge favoured treatment with 2–5-day dose corticosteroids. Although there was no difference between the groups in the risks of ICU admission or death and new or aggravated hypertension, there was a trend towards poorer outcomes in the 14-day dose group. These observations are consistent with previous observational studies that have shown greater risks of infection and mortality when patients are treated with corticosteroids for 10 days rather than 5 days [6, 15]. As in this analysis, the REDUCE trial found that the median length of hospital stay in the 5-day group dose group was 1 day shorter than in the 14-day dose group [13]. Other studies have reached similar conclusions regarding the duration of treatment. A recent meta-analysis compared short-term (≤ 7 days) with longer-term (> 7 days) systemic corticosteroid treatment of patients with exacerbation of severe or very severe COPD and found no differences in the likelihood of treatment failure, risk of relapse, change in lung function tests or adverse effects [16]. Several studies suggest that an even shorter duration of systemic corticosteroid treatment (e.g., 3 days [17], 5 days [13] or 7 days [18]) may be as effective as longer courses in hospitalised patients with AECOPD. These results are similar to those of previous systematic reviews [19] and support the use of shorter systemic corticosteroid treatment regimens.

Corticosteroids are well known for their vast array of side effects both in the short term and over an extended period [20], including new/aggravated diabetes [12], hyperglycaemia, and hypertension [14]. Short-term treatment with systemic corticosteroids has also been associated with an increased risk of bone fractures, venous thromboembolism, and sepsis [9, 21], as well as an increased risk of gastrointestinal bleeding or perforation [22]. Because of these potentially serious adverse effects, it is important to minimise the administration of corticosteroids, particularly for patients with frequent COPD exacerbations. The introduction of biological agents has led to a reduction in systemic corticosteroid use in other disease areas [23], and these agents may also benefit patients with COPD in the future. Emerging data also show that increased levels of blood eosinophils may function as a biomarker for a positive response to corticosteroid treatment. Conversely, when blood eosinophils levels are low, patients with AECOPD may be less likely to respond well to systemic corticosteroid treatment [24–26]. Some evidence suggests that blood eosinophil levels may be measured to reduce corticosteroid use in patients with COPD exacerbations [24, 27]. Importantly, corticosteroid treatment may not benefit patients with exacerbations that are associated with bacterial infections [25, 28]. Microbiome studies have demonstrated that AECOPD treatment with systemic corticosteroids can result in an increased bacterial burden and an increased abundance of specific airway microbiota that persists for several weeks [29]. Taken together, these data do not support the universal prescription of systemic corticosteroids for patients with AECOPD and suggest that this could do more harm than good in some patients.

The strengths of this analysis included its relatively large sample size and the multi-centre RCT design of its constituent studies. It is a strength, that all patients were randomly allocated to a corticosteroid regimen, which eliminates the risk of bias by indication, otherwise a limitation that can be hard to overcome in pooled data; in both the REDUCE and CORTICO-COP trials, the corticosteroid doses administered were subject to randomisation, and not based on the severity of the disease. We used the primary outcome “Days alive and out of hospital within 14 days after recruitment” due to its high sensitivity as an outcome measure, and it was used to report the results of the CORTICO-COP trial. In addition, using this outcome measure avoids lead-time bias since patients who died early would not be counted as having a short length of stay [14, 30]. Systemic corticosteroid therapy seems to shorten the duration of hospital stays compared to placebo. Therefore, we only included studies in which corticosteroids were provided in both trial arms. To test our hypothesis, comparison with a placebo group was unnecessary. Some limitations do, however, need to be considered. We recognize that these two trials were conducted several years apart, and different treatment durations could have explanations other than corticosteroid dose (e.g., the length of hospital admission for an AECOPD may have changed due to better community COPD services). However, the studies are similar in many aspects because there have been few major changes in AECOPD treatment during the years between the trials. Approximately half of the patients in the CORTICO-COP trial were treated with inhaled corticosteroids. We did not have access to the corresponding data for the REDUCE study population; however, because this was a randomised trial, the proportion of patients who received ICS should be similar in the two arms of this trial. Importantly, baseline values differed between the two trials, with REDUCE study patients being more ill. Therefore, it was necessary to adjust for these differences in our analyses (Additional file 1: Table 1). It is also possible that the difference in length of admission and mortality risk between the 14-day regimen and the 2–5-day regimen groups may be attributable not only to corticosteroid dose but also to responsiveness to corticosteroids, which may be influenced by history of asthma, smoking status and blood eosinophil levels. All patients with asthma were carefully excluded from both trials. There was no difference in the number of current smokers between the groups. Unfortunately, there were no blood eosinophil count data from the REDUCE study. Finally, our study may have lacked the necessary power to reliably evaluate the risk of mortality. Therefore, further studies will be necessary to confirm our risk of mortality findings.

## Conclusions

In conclusion, our data do not support using 14-day systemic corticosteroid regimens to treat hospitalised patients with COPD exacerbations. Patients who were randomly assigned to 2–5-day systemic corticosteroid regimens showed more favourable primary outcomes; a result driven by the substantially shorter admission durations. However, we cannot exclude the possibility that these results are due to factors other than corticosteroid dose.

## Supplementary Information

**Additional file 1: Table S1.** Baseline characteristics of study participants from the primary trials. **Table S2.** Analysis of outcomes

## Data Availability

CORTICO-COP data will be available from 1 January 2023, upon request from investigators, if approved by the CORTICO-COP principal investigator and the COP: TRIN Steering Committee. If approved, the data collected for the CORTICO-COP trial, including individual participant data and a data dictionary defining each field in the set, will be made available to others in the form of deidentified participant data. The study protocol and statistical analysis plan for the original study is available at www.coptrin.dk. Informed consent forms will not be available, in accordance with Danish legislation.

## Abbreviations

AECOPD: Acute exacerbation of chronic obstructive pulmonary disease
ICU: Intensive care unit
FEV_1_: Forced expiratory volume in 1 s
FVC: Forced vital capacity
GOLD: Global Initiative for Chronic Obstructive Lung Disease
HR: Hazard ratio
OR: Odds ratio

## References

[1] Singh D, Agusti A, Anzueto A, Barnes PJ, Bourbeau J, Celli BR (2019). Global strategy for the diagnosis, management, and prevention of chronic obstructive lung disease: the GOLD Science Committee Report 2019. Eur Respir J.

[2] Walters JA, Tan DJ, White CJ, Gibson PG, Wood-Baker R, Walters EH (2014). Systemic corticosteroids for acute exacerbations of chronic obstructive pulmonary disease. Cochrane Database Syst Rev.

[3] Niewoehner DE, Erbland ML, Deupree RH, Collins D, Gross NJ, Light RW (1999). Effect of systemic glucocorticoids on exacerbations of chronic obstructive pulmonary disease. Department of Veterans Affairs Cooperative Study Group. N Engl J Med.

[4] Galon J, Franchimont D, Hiroi N, Frey G, Boettner A, Ehrhart-Bornstein M (2002). Gene profiling reveals unknown enhancing and suppressive actions of glucocorticoids on immune cells. FASEB J.

[5] Agusti C, Rano A, Filella X, Gonzalez J, Moreno A, Xaubet A (2003). Pulmonary infiltrates in patients receiving long-term glucocorticoid treatment: etiology, prognostic factors, and associated inflammatory response. Chest.

[6] Sivapalan P, Ingebrigtsen TS, Rasmussen DB, Sorensen R, Rasmussen CM, Jensen CB (2019). COPD exacerbations: the impact of long versus short courses of oral corticosteroids on mortality and pneumonia: nationwide data on 67 000 patients with COPD followed for 12 months. BMJ Open Respir Res.

[7] Borresen SW, Klose M, Baslund B, Rasmussen AK, Hilsted L, Friis-Hansen L (2017). Adrenal insufficiency is seen in more than one-third of patients during ongoing low-dose prednisolone treatment for rheumatoid arthritis. Eur J Endocrinol.

[8] Cain DW, Cidlowski JA (2017). Immune regulation by glucocorticoids. Nat Rev Immunol.

[9] Waljee AK, Rogers MA, Lin P, Singal AG, Stein JD, Marks RM (2017). Short term use of oral corticosteroids and related harms among adults in the United States: population based cohort study. BMJ.

[10] Smit J, Kaasch AJ, Sogaard M, Thomsen RW, Nielsen H, Froslev T (2016). Use of glucocorticoids and risk of community-acquired *Staphylococcus aureus* Bacteremia: a population-based case-control study. Mayo Clin Proc.

[11] Upadhyay J, Trivedi N, Lal A (2020). Risk of future type 2 diabetes mellitus in patients developing steroid-induced hyperglycemia during hospitalization for chronic obstructive pulmonary disease exacerbation. Lung.

[12] Aldibbiat AM, Al-Sharefi A (2020). Do benefits outweigh risks for corticosteroid therapy in acute exacerbation of chronic obstructive pulmonary disease in people with diabetes mellitus?. Int J Chron Obstruct Pulmon Dis.

[13] Leuppi JD, Schuetz P, Bingisser R, Bodmer M, Briel M, Drescher T (2013). Short-term vs conventional glucocorticoid therapy in acute exacerbations of chronic obstructive pulmonary disease: the REDUCE randomized clinical trial. JAMA.

[14] Sivapalan P, Lapperre TS, Janner J, Laub RR, Moberg M, Bech CS (2019). Eosinophil-guided corticosteroid therapy in patients admitted to hospital with COPD exacerbation (CORTICO-COP): a multicentre, randomised, controlled, open-label, non-inferiority trial. Lancet Respir Med.

[15] Groenewegen KH, Schols AM, Wouters EF (2003). Mortality and mortality-related factors after hospitalization for acute exacerbation of COPD. Chest.

[16] Walters JA, Tan DJ, White CJ, Wood-Baker R (2018). Different durations of corticosteroid therapy for exacerbations of chronic obstructive pulmonary disease. Cochrane Database Syst Rev.

[17] Sayiner A, Aytemur ZA, Cirit M, Unsal I (2001). Systemic glucocorticoids in severe exacerbations of COPD. Chest.

[18] Chen G, Xie CM, Luo YF (2008). The effects and therapeutic duration of oral corticosteroids in patients with acute exacerbation of chronic obstructive pulmonary diseases. Zhonghua Jie He He Hu Xi Za Zhi.

[19] Ma Z, Zhang W (2016). Short-term versus longer duration of glucocorticoid therapy for exacerbations of chronic obstructive pulmonary disease. Pulm Pharmacol Ther.

[20] Bleecker ER, Menzies-Gow AN, Price DB, Bourdin A, Sweet S, Martin AL (2020). Systematic Literature Review Of Systemic Corticosteroid Use For Asthma Management. Am J Respir Crit Care Med.

[21] Rogers MAM, Lin P, Nallamothu BK, Kim C, Waljee AK (2018). Longitudinal study of short-term corticosteroid use by working-age adults with diabetes mellitus: risks and mitigating factors. J Diabetes.

[22] Butler E, Moller MH, Cook O, Granholm A, Penketh J, Rygard SL (2019). The effect of systemic corticosteroids on the incidence of gastrointestinal bleeding in critically ill adults: a systematic review with meta-analysis. Intensive Care Med.

[23] Alten R, Nusslein H, Galeazzi M, Lorenz HM, Nurmohamed MT, Bensen WG (2016). Decreased use of glucocorticoids in biological-experienced patients with rheumatoid arthritis who initiated intravenous abatacept: results from the 2-year ACTION study. RMD Open.

[24] Bafadhel M, McKenna S, Terry S, Mistry V, Pancholi M, Venge P (2012). Blood eosinophils to direct corticosteroid treatment of exacerbations of chronic obstructive pulmonary disease: a randomized placebo-controlled trial. Am J Respir Crit Care Med.

[25] Sivapalan P, Jensen JU (2019). Non-eosinophilic severe exacerbations of COPD: what about antibiotics?—authors' reply. Lancet Respir Med.

[26] Bafadhel M, Davies L, Calverley PM, Aaron SD, Brightling CE, Pavord ID (2014). Blood eosinophil guided prednisolone therapy for exacerbations of COPD: a further analysis. Eur Respir J.

[27] Sivapalan P, Lapperre TS, Janner J, Laub RR, Moberg M, Bech CS (2019). Eosinophil-guided corticosteroid therapy in patients admitted to hospital with COPD exacerbation (CORTICO-COP): a multicentre, randomised, controlled, open-label, non-inferiority trial. Lancet Respir Med.

[28] Bafadhel M, McKenna S, Terry S, Mistry V, Reid C, Haldar P (2011). Acute exacerbations of chronic obstructive pulmonary disease: identification of biologic clusters and their biomarkers. Am J Respir Crit Care Med.

[29] Huang YJ, Sethi S, Murphy T, Nariya S, Boushey HA, Lynch SV (2014). Airway microbiome dynamics in exacerbations of chronic obstructive pulmonary disease. J Clin Microbiol.

[30] Ariti CA, Cleland JG, Pocock SJ, Pfeffer MA, Swedberg K, Granger CB (2011). Days alive and out of hospital and the patient journey in patients with heart failure: insights from the candesartan in heart failure: assessment of reduction in mortality and morbidity (CHARM) program. Am Heart J.

