# Supplementary material for: Effect of different corticosteroid regimes for hospitalised patients with exacerbated COPD: pooled analysis of individual participant data from the REDUCE and CORTICO-COP trials
Source: Respir Res. 2021 May 21;22:155. doi: 10.1186/s12931-021-01745-5 (PMC8138920; doi:10.1186/s12931-021-01745-5)
Supplement: Supplementary file 1 — Additional file 1: Table S1. Baseline characteristics of study participants from the primary trials. Table S2. Analysis of outcomes [file 12931_2021_1745_MOESM1_ESM.docx]

Additional Tables

| **Table S1.** Baseline characteristics of study participants from the primary trials | | | | | | |
| --- | --- | --- | --- | --- | --- | --- |
|  | | | **REDUCE** | | **CORTICO-COP** | |
|  |  |  | **Standard**  **treatment**  **(14 days)**  ***n* = 155** | **Short-term treatment**  **(5 days)**  ***n* = 156** | **Standard**  **treatment**  **(5 days)**  ***n* = 159** | **Eosinophil-guided treatment**  **(2 days)**  ***n* = 159** |
| Age in years, Mean (SD) | | | 69.8 (10.6) | 69.8 (11.3) | 75.1 (9.3) | 74.8 (9.2) |
| Women, No. (%) | | | 72 (46.5) | 51 (32.7) | 89 (56) | 86 (54) |
| Index steroid dose in mg, Median (IQR) | | | 560 (560–560) | 200 (200–200) | 230 (230–230) | 117.5 (80–155) |
| Smokers, No. (%) | | |  |  |  |  |
|  | Current | | 62 (40) | 77 (49.4) | 50 (31) | 54 (34) |
|  | Past smoker | | 93 (60) | 79 (50.6) | 105 (66) | 103 (65) |
|  | Never smoked | | 0 (0) | 0 (0) | 4 (3) | 2 (1) |
| Medical Research Council dyspnoea scale, No. (%) | | |  |  |  |  |
|  | 1 | | 4 (2.8) | 4 (2.7) | 3 (1.9) | 5 (3.3) |
|  | 2 | | 14 (9.8) | 13 (8.8) | 9 (5.7) | 15 (9.7) |
|  | 3 | | 15 (10.5) | 23 (15.5) | 52 (32.9) | 41 (26.6) |
|  | 4 | | 43 (30.1) | 45 (30.4) | 50 (31.7) | 49 (31.8) |
|  | 5 | | 67 (46.9) | 63 (42.6) | 44 (27.9) | 44 (28.6) |
| Pre-treatment with antibiotics, No.(%)* | | | 21 (14.0) | 32 (21.9) | 50 (31.5) | 36 (22.6) |
| Pre-treatment with systemic glucocorticoids, No. (%)** | | | 28 (18.5) | 35 (22.6) | 12 (8) | 8 (5) |
| Pack years, Median (IQR) | | | 45 (30–60) | 50 (40–60) | 45 (30–60) | 42 (30–55) |
| FEV_1_ baseline, Median (IQR) % predicted | | | 28 (21–40.8) | 28 (20.1–40.9) | 30 (23.0–40.5) | 32 (23.0–38.5) |
| FEV_1_ day 30, Median (IQR) % predicted | | | 43 (32–60.2) | 43.9 (32.5–64.5) | 39 (27–53) | 40 (27–54) |
| Clinical values, Median (IQR) | | |  |  |  |  |
|  | Blood pressure, mm Hg | |  |  |  |  |
|  |  | Systolic blood pressure | 138 (124–158) | 139 (124–160) | 127 (116–139) | 130 (117–142) |
|  |  | Diastolic blood pressure | 80 (70–87.5) | 80 (71–91) | 70 (62–80) | 70 (62–78) |
|  | Heart rate, beats per min | | 90 (79–105) | 92 (80–106) | 89 (81–99) | 89 (79–99) |
|  | Saturation with supplemental oxygen % | | 95 (92–97) | 95 (92–96) | 95 (93–96) | 95 (93 –97) |

Abbreviations: SD, standard deviation; IQR, interquartile range; FEV_1_, forced expiratory volume in 1 s.

^a^ Based on a linear regression model adjusted for age, sex, smoking status, pre-treatment with antibiotics, pre-treatment with corticosteroids, and the Medical Research Council dyspnoea scale.

^a^ Based on the Cox proportional hazards model adjusted for age, sex, smoking status, pre-treatment with antibiotics, pre-treatment with corticosteroids, and the Medical Research Council dyspnoea scale.

| **Table S2.** Analysis of outcomes | | |  |
| --- | --- | --- | --- |
|  | **5-day regimen** | **14-day regimen** | ***p*-value** |
|  | ***n* = 315** | ***n* = 155** |  |
| **Primary outcome measure** |  |  |  |
| Length of hospital stay ^a^ | Reference | 4.0 (± 0.6) | <0.0001 |
| Mean (±SE) |  |  |  |
| Death during 6-month follow-up period |  |  |  |
| Adjusted hazard ratio (95% confidence interval) ^b^ | Reference | 0.8 (0.4–1.7) | 0.58 |
